# Antimicrobial growth promoters modulate host responses in mice with a defined intestinal microbiota

**DOI:** 10.1038/srep38377

**Published:** 2016-12-08

**Authors:** Kirsty Brown, Sarah J. M. Zaytsoff, Richard R. E. Uwiera, G. Douglas Inglis

**Affiliations:** 1 Agriculture and Agri-Food Canada, 5403-1st Avenue S, Lethbridge, AB, Canada; 2 Department of Agricultural Food and Nutritional Science, University of Alberta, 410 Agriculture/Forestry Centre, Edmonton, AB, Canada

## Abstract

Antibiotics can promote growth in livestock (antimicrobial growth promoters, AGPs), however lack of knowledge regarding mechanisms has hampered the development of effective non-antibiotic alternatives. Antibiotics affect eukaryotic cells at therapeutic concentrations, yet effects of AGPs on host physiology are relatively understudied, partially due to the complexity of host-microorganism interactions within the gastrointestinal tract. To determine the direct effects of AGPs on the host, we generated Altered Schaedler Flora (ASF) mice, and administered chlortetracycline (CTC) and tylosin phosphate (TYL) in feed. Mice were challenged with *Citrobacter rodentium* to determine how AGPs alter host responses to physiological stress. Although CTC and TYL had inconsistent effects on the ASF taxa, AGPs protected mice from weight loss following *C. rodentium* inoculation. Mice treated with either CTC or TYL had lower expression of *βd1* and *Il17a* in the intestine and had a robust induction of *Il17a* and *Il10*. Furthermore, AGP administration resulted in a lower hepatic expression of acute phase proteins (*Saa1, Hp*, and *Cp*) in liver tissue, and ameliorated *C. rodentium*-induced reductions in the expression of genes involved in lipogenesis (*Hmgcl* and *Fabp1*). Collectively, this indicates that AGPs directly affect host physiology, and highlights important considerations in the development of non-antibiotic alternatives.

The beneficial effects of using antibiotics administered at non-therapeutic concentrations in feed as growth promoters (termed antimicrobial growth promoters, AGPs) were first recognized in the 1940’s when chickens fed streptomycin exhibited enhanced growth and feed efficiency^1^. Since then, the growth promoting effects of several classes of antimicrobial agents (AMAs) have been documented in cattle, poultry, and swine via enhanced feed efficiency, prevention of enteric disease, and improved reproductive success^2^. Growing concerns over resistance to AMAs in pathogenic bacteria has resulted in mounting public and government scrutiny regarding AGP use in agriculture^3^,^4^,^5^. As a result, the development of effective non-antibiotic alternatives is critical to maintain the beneficial aspect of AGPs without the risk of selecting for antimicrobial resistance (AMR) in enteric bacteria. Despite AMAs being used in agricultural practices for over 50 years, the mechanisms by which AGPs exert their growth promoting effects is both controversial and relatively unknown^6^,^7^,^8^,^9^.

AGPs are administered in feed, and as such the gastrointestinal tract (GIT) is considered a primary site of AGP action. The physiology of the GIT is extremely complex, involving interactions between dietary and host factors, as well as pathogenic and commensal bacteria. The intestines are colonized by complex microbial communities which are implicated in maintaining the balance between health and disease both in the intestines and in organs throughout the body^10^. Early studies indicated that germ-free mice do not exhibit growth promoting effects from AGPs^11^, which promulgated the belief that the intestinal microbiota was central to the AGP mechanism of action^12^. The ‘microorganism-centric hypothesis’ proposes that AGP-induced changes in the microbiota lead to enhanced growth by shifting the microbiota thereby decreasing competition for nutrients, preventing pathogen colonization, and enhancing microorganisms that can extract more energy from the diet^9^,^12^.

The direct effects of AGPs on host physiology have not been extensively studied. The intestine not only functions as the site of nutrient absorption but is a major immunologically responsive organ. The ‘host-centric hypothesis’ is supported by evidence that several AMAs have immune-modulating properties at therapeutic concentrations, which include down-regulation of prolonged inflammation, increased mucous clearance, and modified phagocyte activity^13^. The immune-modulating effects of several classes of AMAs have been noted in cell culture^14^,^15^,^16^, and in agnotobiotic murine models non-therapeutic concentrations of AMA modulate intestinal inflammation^6^, and increase hepatic metabolism leading to adiposity^17^.

Understanding the mechanisms of AGP action are hindered by the complex interactions among AMAs, bacteria, and the host within the GIT. In the current study, a reductionist mouse model (i.e. Altered Schaedler Flora mice) was used to examine the direct effects on the host while reducing the potential confounding effects of AMA administered at non-therapeutic concentrations on a conventional enteric microbiota. The Altered Schaedler Flora (ASF) is known to normalize the deficits in intestinal development that are observed in germ-free mice, namely restoration of cecal size and function^18^. ASF mice were administered two commonly used agricultural AGPs (chlortetracycline and tylosin phosphate) representing tetracycline and macrolide antibiotic families, respectively. *Citrobacter rodentium* is a murine enteric pathogen that shares characteristics of pathogenesis with human enteropathogenic and enterohaemorrhagic *E. coli*^19^. To induce physiological stress within the intestine and identify alterations in responses, the murine enteropathogen *C. rodentium* was used as an inflammation incitant.

## Results

### CTC and TYL administration enhanced feed consumption and ameliorated weight loss in infected mice

**Figure 1:**
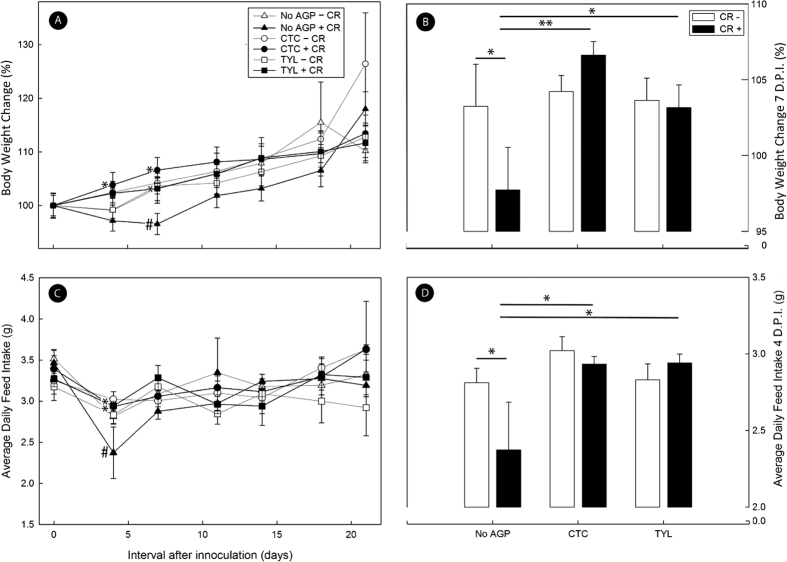
Effect of AGP treatment on body weight and feed intake. Mice were inoculated with *C. rodentium* (CR+) or PBS (CR−) that received feed without AGPs (CTRL), feed with 5.5 mg of chlortetracycline/kg (CTC), or feed with 22 mg tylosin phosphate/kg (TYL). (**A**) Change in body weight (%) throughout the disease process. (**B**) Change in body weight (%) at peak infection (i.e. 7 days p.i.). (**C**) Average daily feed intake (g) throughout the disease process. (**D**) Average daily feed intake at 4 days p.i. Bars represent mean ± standard error of the mean (SEM, n = 3–4). *P < 0.05 for CR+ and No AGP treatment mice relative to other treatments. ^#^P < 0.05 CR− and CTRL treatment mice relative to other treatments.

Infected (CR+) but not uninfected (CR−) mice that that were not administered AGPs exhibited a lower body weight (p = 0.016) and feed consumption (p = 0.026) at peak infection (i.e. ca. 7 days post inoculation (p.i.)) (Fig. 1A–D). CR+ mice administered either CTC or TYL in feed consumed more food (p ≤ 0.029) than CR+ mice not administered AGP, and weight gain in CR+ mice administered AGPs was equivalent (p ≥ 0.088) to CR− mice regardless of AGP treatment.

### AGPs did not affect densities of *C. rodentium*

**Figure 2:**
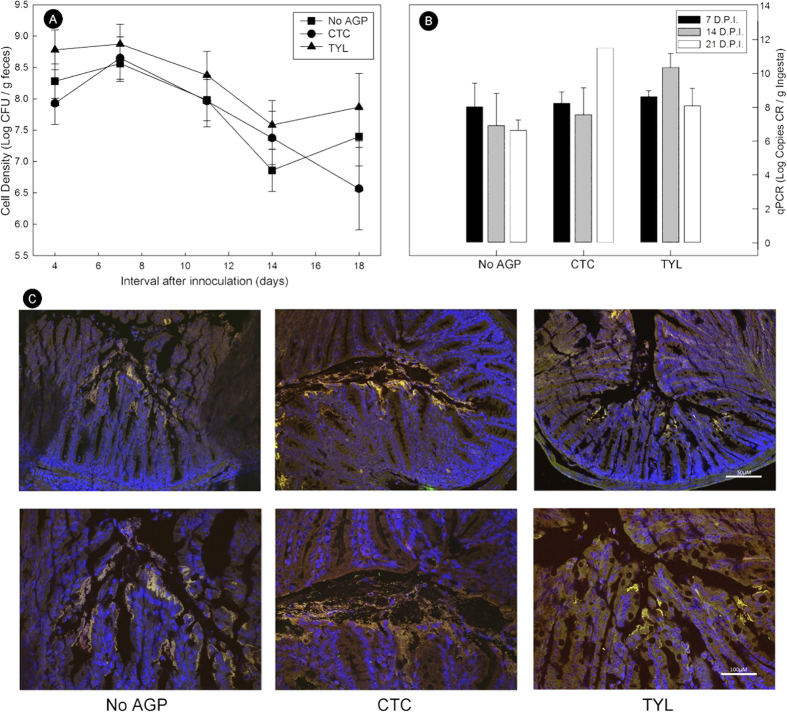
Densities of *Citrobacter rodentium* associated with mice. Animals were inoculated with *C. rodentium* (CR+) or PBS (CR−) that received feed without AGPs (CNRL), 5.5 mg of chlortetracycline/kg of feed (CTC), or 22 mg tylosin phosphate/kg feed (TYL). (**A**) Densities of live *C. rodentium* (log CFU/g) in feces as determined by dilution plating. (**B**) Densities of *C. rodentium* (log copies/g) in colonic ingesta at 7, 14, and 21 days p.i. as determined by quantitative PCR. Vertical lines associated with markers and histogram bars the SEM (n = 3–4). (**C**) Visualization of *C. rodentium* in colon tissues at peak infection (i.e. 7 days p.i.); cell nuclei are stained with DAPI (blue), total bacteria are stained with Alexa-488 (green) and γ-proteobacteria (*C. rodentium*) are stained with Alexa-594 (red). Scale bar = 50 μm (20X images) and 100 μm (40X images).

Administration of CTC or TYL did not affect (p = 0.084) the abundance of *C. rodentium* shed in feces of CR+ mice throughout study period (Fig. 2A), nor densities of the bacterium associated with colonic mucosa (p = 0.141) (Fig. 2B). Examination of colonic tissues by fluorescent *in situ* hybridization in CR+ mice confirmed the equality of *C. rodentium* abundance, and spatial distributions of the bacterium in association with mucosa (Fig. 2C).

### TYL and CTC differentially affect Altered Schaedler Flora taxa in mice

**Figure 3:**
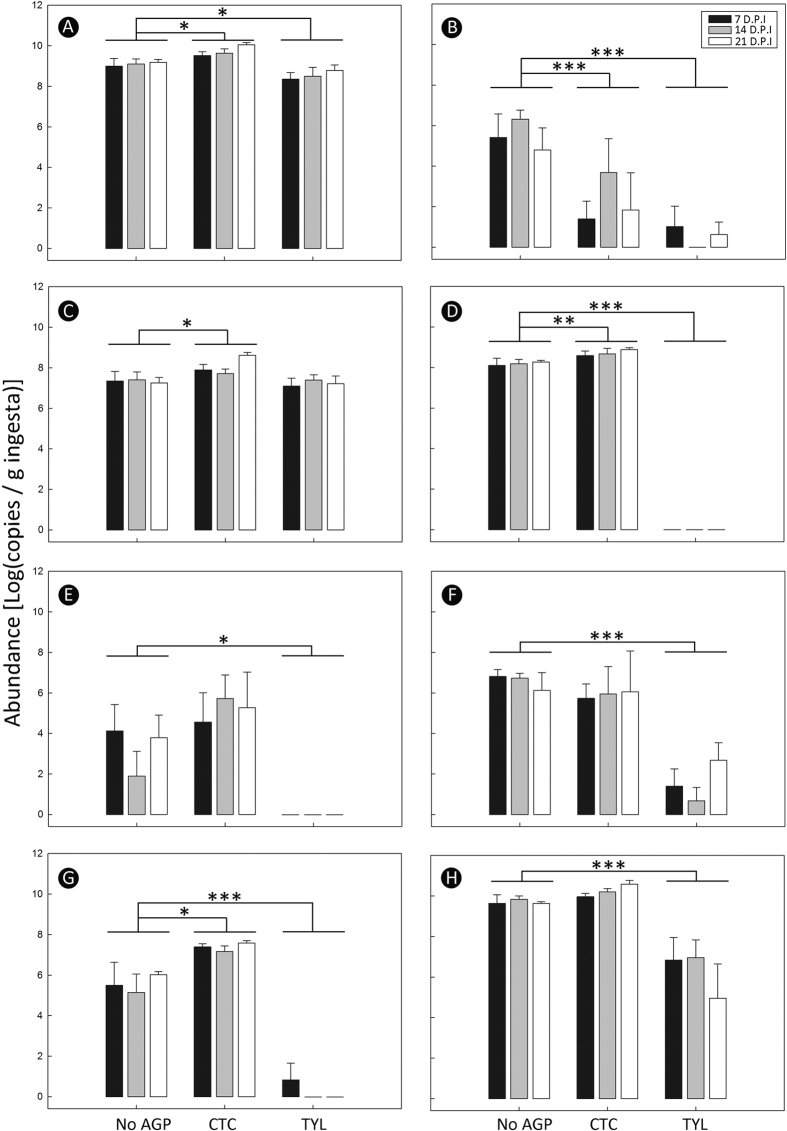
Abundance of bacteria (log copies/g) in colonic ingesta. Mice received feed without AGPs (CNRL), 5.5 mg of chlortetracycline/kg of feed (CTC), or 22 mg tylosin phosphate/kg feed (TYL). (**A**) Total bacteria. (**B**) *Clostridium* gp. (ASF356). (**C**) *Lactobacillus murinus* (ASF361). (**D**) *Flexistipes* gp. (ASF457). (**E**) *Eubacterium plexicaudatum* (ASF492). (**F**) Low GC content Gram + gp. (ASF500). (**G**) *Clostridium* gp. (ASF502). (**H**) *Bacteroides* sp. (ASF519). Luminal contents were processed at 7, 14 and 21 days p.i. Bars represent mean ± SEM (n = 7–8). *P < 0.05, **P < 0.01, ***P < 0.001.

Densities of ASF bacteria in colonic digesta of CR− and CR+ mice ranged from 8.4 ± 0.33 to 10.1 ± 0.10 log_10_ copies/g (Fig. 3A). Total bacterial densities were higher (p = 0.020) in mice administered CTC, and this was attributed to higher (p ≤ 0.014) densities of ASF361 (*Lactobacillus murinus*), ASF457 (*Flexistipes* gp. *Eubacterium*) and ASF502 (*Clostridium* gp.) (Fig. 3C,D,E,G). Total bacteria densities were lower (p = 0.045) in mice administered TYL (Fig. 3A). In mice administered TYL, densities of ASF356 (*Clostridium* sp.), ASF500 (low GC content gram positive group), ASF502 (*Clostridium* sp. 2), and ASF519 (*Bacteroides* sp.) were lower (p ≤ 0.001), and ASF457 (*Flexistipes* sp.) and ASF492 (*E. plexicaudatum*) were eliminated (Fig. 3B,D–H). Only ASF361 (*L. murinus*) was not impacted by TYL administration. The administration of TYL had considerable impacts on the densities of several ASF taxa; however, CTC exerted minimal effects. Notably, TYL and CTC had varying effects and did not alter ASF taxa in a similar manner.

### CTC and TYL alter expression of genes that regulate inflammatory responses in the intestine

**Figure 4:**
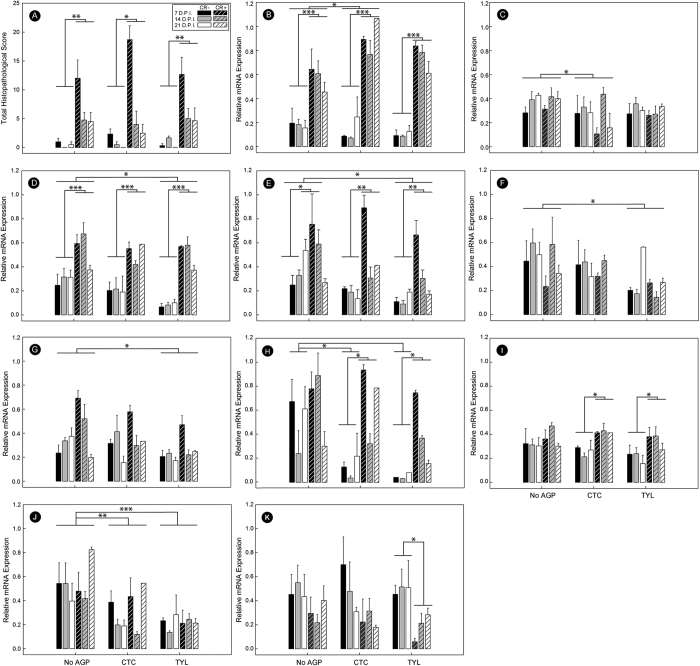
Histopathological responses and expression of inflammatory and host defence genes in the colonic mucosa. Mice were inoculated with *C. rodentium* (CR+) or PBS (CR−), received feed without AGPs (CTRL), feed with 5.5 mg of chlortetracycline/kg (CTC), or feed with 22 mg tylosin phosphate/kg (TYL) and colons were processed at 7, 14, and 21 days p.i. (**A**) Average total histopathological scores (maximum possible score of 24). (**B–K**) Relative expression of mRNA transcripts. (**B**) *Tnfα*. (**C**) *Tgfβ*. (**D**) *Ifnγ*. (**E**) *Il1β*. (**F**) *Il4*. (**G**) *Il6*. (**H**) *Il17a*. (**I**) *Il10*. (**J**) *βd1*. (**K**) *Cramp*. Bars represent mean ± SEM (n = 3–4). *P < 0.05, **P < 0.01, and ***P < 0.001.

Although higher histopathologic scores (p < 0.001) were observed in CR+ relative to CR− mice, AGP administration did not affect (p ≥ 0.294) histopathological scores of intestinal damage (Fig. 4A). However, AGPs administered in feed to mice modulated key molecular metrics of inflammation (Fig. 4B–K). Mice that received CTC exhibited higher expression (p = 0.014) of the Th1 pro-inflammatory cytokine, tumor necrosis factor-α (*Tnfα*), and lower (p = 0.009) expression of the anti-inflammatory cytokine transforming growth factor-β (*Tgfβ1*) (Fig. 4B). Mice administered TYL exhibited lower expression of the Th1 cytokine, interferon-γ (*Ifnγ*) (p = 0.003), the Th17 cytokines, interleukin-1β (*Il1β*) (p = 0.002) and interleukin-6 (*Il6)* (p = 0.002), and the Th2 cytokine, interleukin-4 (*Il4*) (p = 0.003) (Fig. 4D–G). Administration of both CTC and TYL was associated with a lower (p ≤ 0.007) expression of the Th17 cytokine, interleukin-17a (*Il17a*) in CR+ mice (Fig. 4H). The expression of *Il17a* in CR+ mice was elevated (p ≤ 0.047) relative to non-infected CTC and TYL, but not CTRL treatment mice. Although there was no difference (p = 0.395) in expression of the T_reg_ cytokine, interleukin-10 (*Il10*) between the AGP treatments, expression of *Il10* gene mRNA was suppressed (p ≤ 0.002) in CR− mice administered CTC and TYL relative to CR+ mice (Fig. 4I). Furthermore, expression of the antimicrobial peptide, β-defensin 1 (*βd1*) gene was lower in mice administered CTC (p = 0.031) and TYL (p < 0.001) (Fig. 4J). In CR+ mice, the expression of cathelicidin related antimicrobial peptide (*Cramp*) was suppressed (P = 0.019) in animals administered TYL, with a similar trend (p = 0.059) observed in CTC mice (Fig. 4K).

### Systemic immunomodulatory effects of AGPs in response to *C. rodentium* infection

**Figure 5:**
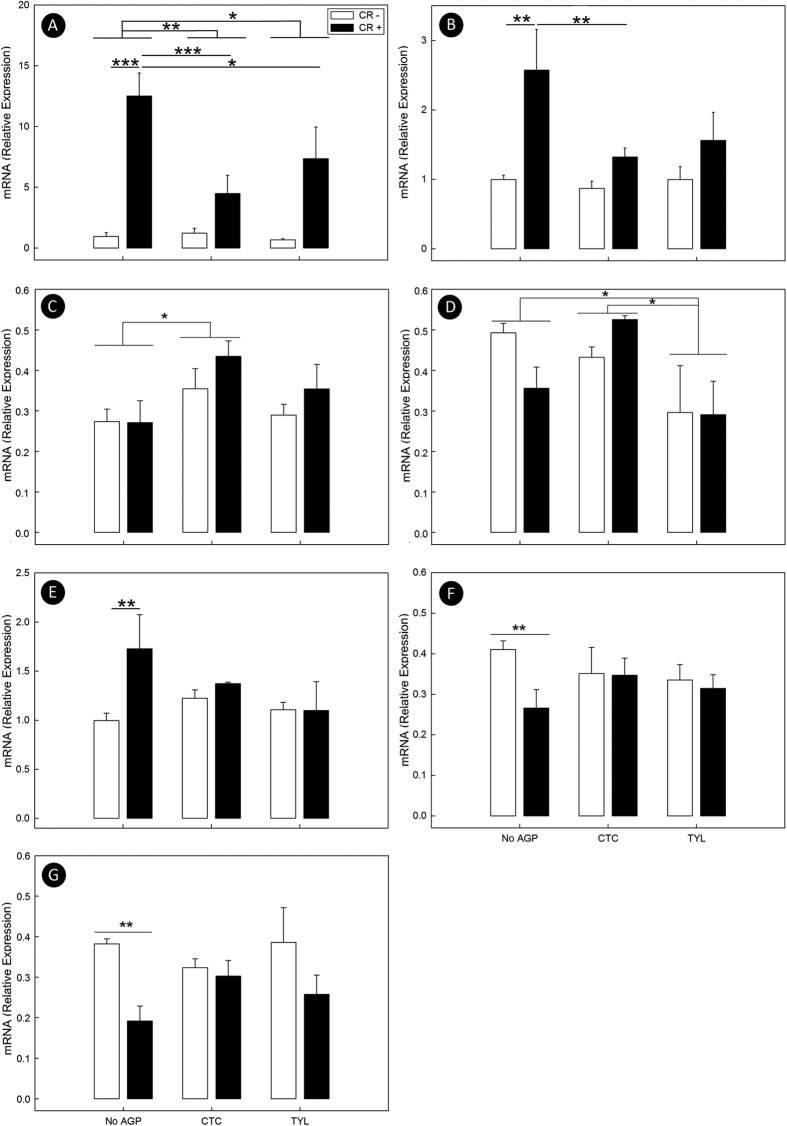
Expression of liver mRNA transcripts involved in lipogenesis and acute phase responses. Mice were inoculated with *C. rodentium* (CR+) or PBS (CR−), received feed without AGPs (CTRL), feed with 5.5 mg of chlortetracycline/kg (CTC), or feed with 22 mg tylosin phosphate/kg (TYL), and livers were processed at peak infection (i.e. 7 days p.i.). (**A**) *Saa1*. (**B**) *Hp*. (**C**) *Cp*. (**D**) *Hmgcl*. (**E**) *Fabp1*. Bars represent mean ± SEM (n = 3–4). *P < 0.05, **P < 0.01, ***P < 0.001.

Liver tissue was analyzed throughout the experiment to evaluate the induction of systemic inflammation and immune-modulation. At 14 and 21 days p.i. no effect of AGP on expression of acute phase proteins (APPs) was observed (*data not presented*). In contrast, at peak infection (i.e. 7 days p.i.), expression of mRNA encoding for acute phase protein serum amyloid A1 (*Saa1*) was lower (p ≤ 0.044) in mice administered both CTC and TYL (Fig. 5A). CR+ mice exhibited higher (p ≤ 0.018) expression of *Saa1*, as well as haptoglobin (*Hp*) and ceruloplasmin (*Cp*), but this effect was absent in mice that were administered CTC or TYL (Fig. 5A–C). Furthermore, CR+ mice administered both CTC and TYL displayed lower (p ≤ 0.024) expression of *Saa1* compared to CR- mice (Fig. 5A). CR+ mice administered CTC also exhibited lower (p = 0.021) expression of *Hp*, with a similar trend (p = 0.053) observed in CR+ mice administered TYL (Fig. 5A,B).

### AGPs protect against infection-induced changes in expression of hepatic metabolic genes

In addition to the production of APPs, the liver also plays an important role in lipid metabolism. Thus, the expression of genes involved in lipid metabolism were analyzed. There were no effects of AGPs (p ≥ 0.167) on the expression of hepatic metabolic genes following AGP treatments at 14 and 21 days p.i. Furthermore, AGP treatment did not affect (p = 0.301) triglyceride content in livers at 7 days p.i. (*data not presented*). However, higher (p = 0.016) citrate transport protein 1 (*Ctp1*) expression was observed in mice administered CTC, and a lower (p = 0.042) level of apolipoprotein A2 (*ApoA2*) expression was observed in TYL treatment mice (Fig. 5D,E). In addition, the expression of the 3-hydroxymethyl-3-methylglutaryl-CoA lyase (*Hmgcl*) and the fatty acid binding protein 1 (*Fabp1*) genes was lower (p ≤ 0.017) in CR+ mice not administered AGPs (Fig. 5D,E). In contrast, there was no difference (p ≥ 0.066) in expression of *Hmgcl* and *Fabp1* in liver tissue between CR+ and CR− mice that were administered either CTC or TYL.

## Discussion

Despite the considerable health benefits that AGPs confer on livestock, the biological mechanisms by which AGPs function are poorly understood, in large part as a result of the complex interactions between the microbiome and the host intestine^20^. To address this, we examined the effects of two model AGPs, CTC and TYL, on host immune function and metabolism in gnotobiotic mice with a simplified microbiota containing eight taxa. *Citrobacter rodentium* infection was used to incite immunological and physiological stress in mice in order to identify how AGPs alter host responses. Neither CTC nor TYL decreased the level of colonization by *C. rodentium* indicating that the antibiotics were below the minimum inhibitory concentration for the enteric pathogen. Despite similar levels of colonization with *C. rodentium*, mice administered either CTC or TYL did not experience weight loss in the early stages of infection. In general, a mouse consumes ≈1.5 g of feed per 10 g body weight per day and gradual weight gain is expected until ≈7 weeks of age^21^. As such, a reduction in feed intake and lower weight in *C. rodentium*-challenged mice is consistent with physiological distress, necessitating that the animal directs its metabolic resources on clearing the infection rather than anabolic functions that include weight gain. Although AGPs did not alter the ability of *C. rodentium* to colonize mice, both CTC and TYL may alter physiologic and inflammatory responses in the GIT resulting in decreased severity of pathogenesis and subsequent improvement in weight gain and feed consumption.

The effects of AGPs on the intestinal microbiota have been documented in a number of animal studies, although results are variable. Several investigations in broiler chickens indicated that the community structure was altered by AGP treatment concurrent with increased body weight^22^,^23^,^24^. Conversely, other studies in beef cattle, chickens, and pigs concluded that bacterial communities were not altered by AGP treatment^25^,^26^,^27^, although variations in individual taxa were observed in some of these studies^26^,^28^,^29^,^30^,^31^. The differing effects of AGP may be attributed, at least in part, to the complexity of the microbial communities within the GIT in the different animal species and complex interactions between intestinal bacteria and the host. Germ-free mice have known deficits in intestinal immune function^32^ and although ASF taxa were originally selected on their ability to normalize the enlarged cecum observed in germ-free mice^33^, more current research has shown that ASF mice are immunologically, reproductively and metabolically similar to mice with a conventional microbiota^18^. Thus, ASF mice represent an appropriate model to study the effects of AGPs on known commensal microorganism while also assessing effects on the host that exhibits normalized immune and metabolic responses. Importantly, a growing body of evidence has demonstrated that the composition of the microbiota is critical in directing particular immune responses within the intestine^34^, and thus bacteria that comprise the ASF may play a role in the induction and progression of inflammatory processes within the host. We found that non-therapeutic levels of TYL and to a lesser extent CTC altered the densities of specific ASF taxa in the colon, but individual taxa were not affected equally by these two AGPs. Although overall bacterial load and the density of certain microbial taxa were higher in mice administered CTC, TYL treatment was associated with lower bacterial load and density of individual taxa. Importantly, ASF communities were differentially altered by CTC and TYL. These data indicate that AGPs can alter bacterial populations within the intestine, albeit most likely by different mechanisms.

The predominant theory of AGP function is a bacteria-centric process; that AGPs cause shifts in the bacterial community composition within the intestine resulting in a more efficient system for nutrient uptake. This may include decreased competition for nutrients, prevention of pathogen colonization, and/or selecting for bacteria that are better able to extract more energy from the diet^9^. However, no single bacterium or group of bacteria has been identified that is linked to enhanced growth following treatment with AGPs^20^. It is well known that antibiotics administered at therapeutic concentrations can affect the host by directly modulating immune function and reducing inflammation^13^, and this has stimulated the emergence of host-centric (immunomodulation) hypotheses. The immunomodulation hypothesis suggests that AGPs dampen physiological inflammation in the intestinal mucosa, decreasing the catabolic costs to the host of maintaining an immune response thereby allowing more nutritional resources to be dedicated to anabolic processes involved in growth^6^,^7^. Although the majority of studies conducted to date have examined the physiological effects of AMAs at therapeutic concentrations, a limited number of studies have examined host effects of AMAs administered at non-therapeutic concentrations^20^. For example, the administration of vancomycin and penicillin at non-therapeutic doses to mice increased adiposity by altering hepatic metabolism of lipids and cholesterol^17^,^35^. Furthermore, a non-therapeutic dose of CTC orally administered to mice regulated intestinal inflammation and altered responses to *C. rodentium*^6^. More recent studies have examined physiological changes in pigs following the administration of AGPs in feed, and lower *Ifn-γ* expression and fewer CD4+ T cells were reported in the serum of pigs treated with chlortetracycline^36^ as well as downregulation of serum proteins relating to inflammation, oxidation and lipid metabolism in those administered oxytetracycline^37^. Consistent with these findings, we observed differential expression of inflammatory markers in ASF mice administered AGPs. Indeed, mice administered CTC demonstrated higher expression of a single Th1 cytokine (*Tnfα*) and lower expression of the anti-inflammatory and reparative cytokine, *Tgfβ*. Moreover, ASF mice administered TYL in feed showed lower expression of Th1 (*Ifnγ, Il1β*), Th2 (*Il4*), and Th17 (*Il6*) cytokines. Expression of Th17 (*Il17a)* and Th2 (Il10) cytokines, as well as defensins (*βd1)* were similarly deregulated by both CTC and TYL in ASF mice.

The fluctuations in inflammatory cytokine profiles are important for the induction of pro- and anti-inflammatory processes, a requirement for both clearing infections and healing injured tissue. In the context of our research, however, the interplay between pro- and anti-inflammatory signals also impacts the balance between catabolic-anabolic processes and subsequently affect host growth. In the current study we observed early changes in Th17 cytokine, *Il17a*. It is known that Th17 T cells are initiated by adhesion of commensal or pathogenic bacteria to epithelial cells^38^. Thus, it is plausible that the decreased expression of Th17 cytokines observed in CR-ASF mice treated with CTC and TYL indicates that AGPs are involved in mitigating interactions between pathogens and the host; an important function to prevent pathogen invasion of the intestinal mucosa^39^. In addition, mice that were administered AGPs exhibited lower expression of the antimicrobial peptide, *βd1* that is also involved in regulating host-bacterial interactions by exhibiting antimicrobial activity against a broad range of organisms^40^,^41^, particularly Gram-positive bacteria^42^. Following infection with *C. rodentium*, ASF mice treated with CTC and TYL displayed higher expression of Th17- and Th2-associated cytokines (i.e. *Il17* and *Il10*) that are involved in the clearance and resolution of *C. rodentium* infection^43^. This may indicate that AGPs are causing a dampened inflammatory status under basal physiological conditions, but allows a robust inflammatory response under conditions of infection. While studies have shown the effects of individual AMAs on the host, our investigation demonstrates that changes in expression of similar bioactive markers in CTC and TYL treated mice may indicate common mechanisms of action for AGPs.

Blood from the intestine flows to the liver, and hepatocytes have a critical function in regulation of both metabolism and the induction of inflammatory and immune responses associated with products from portal blood^44^. While emerging evidence supports that AGPs impart an immunomodulatory effect in the GIT, the immunomodulatory effects of AGPs in the liver are undetermined at present. We observed that mice that were administered either CTC or TYL at non-therapeutic doses exhibited lowered expression of several APPs, and these mice were protected from induction of APPs (i.e. as observed in mice infected with *C. rodentium*). Following inoculation with *C. rodentium*, mice that were not administered AGPs exhibited higher expression of *Saa1, Hp*, and *Cp*. Yet mice that were administered either CTC or TYL did not display a strong APP induction response. Serum amyloid A1 is induced by cytokines (*Il1β, Il6, Tnfα*), and is proposed to have downstream effects on cytokine/chemokine induction, chemotaxis, and release of inflammatory mediators through activation of multiple receptors including TLR2, TLR4, FPR2, SR-BI, and P2X7^45^,^46^. Haptoglobin has anti-oxidant function which prevents tissue damage as a result of inflammation^47^,^48^, and ceruloplasmin oxidizes Fe(II) to Fe(III) and is proposed to dampen inflammation by protecting against oxidizing compounds such as neutrophil released myeloperoxidase^49^. The decrease in both the inflammatory and resolution aspects of the APP response in mice administered CTC and TYL at non-therapeutic doses indicates that AGPs may have protective effects by dampening inflammation in the liver.

In addition to its role in the APP response, the liver is responsible for the distribution of nutrients to tissues throughout the body. The administration of antibiotics (e.g. vancomycin and penicillin) at therapeutic doses has been shown to downregulate lipid metabolism^17^, suggesting that antibiotics can modulate metabolic functions of the liver. Furthermore, antibiotic exposure early in life has been associated with obesogenic phenotypes in both mice and humans^35^,^50^. However, the connection between inflammation and metabolism in the liver is largely undefined. We showed CTC caused increased expression of *Cpt1a*, an important component in β oxidation of long chain fatty acids. Furthermore, TYL administration was associated with a lowered expression of *ApoA2* a protein that appears to be involved in cholesterol and HDL metabolism, and imparts anti-inflammatory and anti-oxidative effects as well^51^. Collectively these observations indicate that AGP may deregulate liver lipid metabolism, an unexpected and perhaps undesirable consequence of AGP use. We showed that under conditions of immunological stress following *C. rodentium* challenge, mice not treated with AGP had decreased expression of 3-hydroxymethyl-3-methylglutaryl-CoA lyase (*Hmgcl*) and fatty acid binding protein 1 (*Fabp1*) both of which are involved in lipogenesis^52^,^53^. However, CR+ mice administered either CTC and TYL did not exhibit decreased expression of these metabolic genes. This suggests that in addition to immune-modulation, AGPs may protect the host from metabolic deregulation that occurs following infection.

The reductionist ASF mouse model used in the current study facilitated the elucidation of AMA effects on the host. Our data indicates that while CTC and TYL modulated the simplified microbiota to differing extents, certain host responses to immunological stress in the intestines and liver were similarly modulated by both AGPs. Thus, it appears that the growth promoting characteristics of AGPs are not exclusively a ‘bacteria-centric’ or ‘host-centric’ process, but rather a harmonization of both aspects. It also cannot be ruled out that changes in bacterial populations may be a result of AGP-induced changes in host responses. Our findings also indicate that AMAs administered at non-therapeutic doses not only affect intestinal function, but also the function of key extra-intestinal tissues such as the liver. In conclusion, our findings underscore the importance of considering both the intestinal microbiota, as well as host physiology and immunology when investigating the effects of AGPs on growth. Furthermore, our work demonstrates that AMAs administered at non-therapeutic doses affect a variety of biological systems within the host, including potentially detrimental sequelae for the health of livestock fed AGPs as well as people exposed to low levels of AMAs. Finally, our study provides fundamental information that will facilitate the elucidation of the mechanisms by which AGPs promote growth toward developing efficacious and non-antibiotic alternatives to AGPs for use in livestock production.

## Methods

### Experimental design

The experiment was arranged as a completely randomized design with three levels of AGP treatment, two levels of immunological stress, and three levels of time p.i. Each replicate included 18 mice, and four replicates were conducted on separate occasions (72 animals in total).

### Ethics statement

The study was carried out in strict accordance with the recommendations specified in the Canadian Council on Animal Care Guidelines. The project was reviewed and approved by the Lethbridge Research and Development Centre (LRDC) Animal Care Committee (Animal Use Protocol Review 1307), and the LRDC Biosafety and Biosecurity Committee before commencement of the research.

### Mice

A gnotobiotic ASF colony was generated at the small animal facility located at LRDC using donor ASF mice (Taconic, Germantown, NY, USA) to passively transmit the ASF taxa to germ-free mice^54^. The resulting female and male ASF adult mice were maintained in a germ-free isolator (Class Biologically Clean, Madison, WI, USA) in a 12 hr light/dark cycle, and allowed to drink sterilize food (93 G Purified Rodent, Canadian Lab Diets, Leduc, AB) and water *ad libitum*. The presence of ASF taxa in breeder mice was confirmed in feces as described below for digesta. In addition, the gnotobiotic status of breeder mice was confirmed by terminal restriction fragment length polymorphism analysis of feces^55^,^56^. ASF adults were paired to produce ASF offspring. Weaned adolescent mice (3 weeks of age) were aseptically transferred to individually ventilated cages (IVC; Techniplast, Montreal, QC) connected to a HEPA-filtered controller, and maintained under containment mode for the duration of the experimental period. Mice were maintained in a 12 hr light/dark cycle, and fed sterilized food and water *ad libitum* as above.

### Antimicrobial growth promoters

The three AGP treatments were: (i) CTRL (i.e. no AGPs); (ii) chlortetracycline (CTC); and (iii) tylosin phosphate (TYL). For correlation to the amount of AGP use in agricultural industry, the administration rate of CTC (5.5 mg of CTC/kg of feed) recommended by Alpharma Canada Corporation for growth rate and feed efficiency enhancement in swine was used. Similarity, TYL was administered at 20.2 mg TYL/kg of feed based on recommendation by Elanco Animal Health to “increase rate of weight gain and improve feed efficiency”. The body weights for female and male C57BL/6 mice by age (weeks) were obtained from Charles River Laboratories International, Inc. (Wilmington, MA) and the average weight across gender was calculated. Food consumption rates for female and male C57BL/6 were calculated as 1.5 g of food consumed per day per 10 g body weight^21^,^57^. An average food consumption value across gender was calculated, and amounts of CTC and TYL for mice were adjusted to account for the differential feed intake, weight, and weight gain of mice according to the strategy previously employed in rodents^58^. The final amounts of CTC and TYL fed to mice were 5.5 g/kg and 20.2 g/kg, respectively. Aureomycin 110 G (Alpharma Inc., Mississauga, ON) and Tylan 40 (Elanco Animal Health, Guelph, ON) were used as a source of AGPs, and were incorporated into the diet by Dyets Inc. (Bethlehem, PA) based on concentrations of active ingredient within the formulations (11.0% CTC, and 8.8% TYL). The three diet treatments (i.e. CTRL, CTC, TYL) were sterilized by gamma irradiation and beginning at 3-weeks of age, the mice were fed the three diets *ad libitum* throughout the experimental period.

### *Citrobacter rodentium* inoculation

To produce inoculum, *C. rodentium* was grown in Luria-Bertani broth for 16 hr at 37 °C. The bacterial suspension was centrifuged for 5 min at 1600 × g, and the pelleted cells were re-suspended in sterile phosphate buffered saline (pH 7.2; PBS) to a final bacterial concentration of 10^9^ colony-forming units (CFU)/mL. At 6 weeks of age, mice were either gavaged with 100 μL of *C. rodentium* (10^8^ CFU total) or PBS (CR−) for two consecutive days.

### Isolation of *Citrobacter rodentium* from feces

Fresh feces was collected at 3–4 day intervals, weighed, and a subsample was homogenized in 1.0 mL Columbia broth (Himedia, VWR, Edmonton, AB, Canada) within 2 hr of collection. The homogenate was diluted in a 10-fold dilution series, and 100 μL aliquots of each dilution was spread on MacConkey agar (Becton, Dickinson and Company, Mississauga, ON). Cultures were incubated at 37 °C for 24 hr, and colonies of *C. rodentium* were enumerated at the dilution yielding 30 to 300 CFU per dish, and adjusted by the initial weight of the sample.

### Animal euthanization and sample collection

Randomly-selected mice were euthanized on days 7, 14, and 21 p.i., corresponding to early, peak, and late stages of infection, respectively. Mice were anesthetised with isoflurane and then euthanized by cervical dislocation. Immediately after death, a mid-line laparotomy was used to exteriorize the intestine, and a gross pathological assessment of the intestine and associated tissues was completed. The intestine and liver were harvested aseptically. Segments of the distal colon and cecum were: (i) weighed and processed for enumeration of *C. rodentium* CFU as above; (ii) weighed and frozen at −80 °C for DNA extraction and analysis of bacterial communities; and (iii) fixed in 10% neutral buffered formalin for histopathologic and fluorescent *in situ* hybridization analyses. Tissue samples (liver and distal colon) were also placed in RNAlater (Qiagen Inc., Toronto, ON) and stored at −20 °C for mRNA extraction.

### Altered Schaedler Flora community characterization

Genomic DNA from the luminal content of colonic sections was extracted using a DNA Stool kit (Qiagen Inc.). Genomic DNA extracted from pure cultures of individual bacteria that comprise the ASF were used as standards; DNA was extracted using DNA Stool kit (Qiagen Inc.) and standards were generated by amplifying species specific 16S gene segments from the ASF taxa using specific primers^59^. Amplicon DNA was quantified fluorometrically (Qubit, Life Technologies, Burlington, ON, Canada) and copies of gene targets were normalized to 10^7^ copies/μL based on DNA concentration, amplicon size, and nucleotide weight. Copies of ASF bacteria in luminal content of mice was determined relative to the standard curve of known concentration and normalized based on the initial weight of the sample.

### Histopathology

Tissue samples were fixed in 10% buffered formalin for 24 hr, dehydrated, and embedded in paraffin blocks, sectioned (5 μm), de-paraffinized with xylene, and stained with hematoxylin and eosin. Tissues were scored by a veterinary pathologist (R.R.E.U.) blinded to the treatments using a modified scoring criteria described previously^6^,^60^. Colonic sections were graded 0 to 4 for epithelial cell hyperplasia, crypt height, epithelial injury, extent of inflammatory infiltrates, and 0 to 3 for mitotic activity of epithelial cells, and goblet cell depletion. The total pathology score was obtained by calculating the sum of scores for all categories for each mouse.

### Quantification of mRNA expression in colon and liver tissue

Total RNA was extracted from colon and liver tissues using the RNeasy mini kit (Qiagen Inc.) according to manufacturer’s instructions with the addition of a DNase step to remove residual genomic DNA. RNA quality and quantity was determined using Bioanalyzer RNA 6000 Nano kit (Agilent, Mississauga, ON, Canada), and 1 μg of RNA was reverse transcribed to cDNA using Quantitect reverse transcription kit (Qiagen Inc.). Quantitative PCR was performed on an ABI7900HT instrument (Applied Biosystems, Carlsbad, CA) using Quantitect SYBR green master mix (Qiagen Inc.) with the following PCR conditions: 95 °C for 15 min, 40 cycles of 95 °C for 15 sec, 58 °C for 30 sec, and 72 °C for 30 sec, followed by a melt curve analysis from 55–95 °C. cDNA primer sequences specific to gene targets (Table 1) were generated using NCBI primerBLAST; primers were designed to span an exon junction, create a amplicon between 75 and 200 base pairs, and have a melting temperature within 3 °C of 58 °C. Primers were checked against the mouse genome and were required to have at least two mismatches within the last five base pairs at the 3′ end of the primer. Primer efficiency was between 85–115% and a single peak was present in melt analysis. qPCR reactions were run in triplicate and the average cT values were used to calculate gene expression relative to three reference genes (*Hprt, Ppia*, and *Gusβ*) using qBase+ software (Biogazelle, Gent, Belgium) as per the minimum information for publication of quantitative real-time PCR experiment guidelines^61^.

### Fluorescence *in situ* hybridization

Fixed paraffin embedded colon section were deparaffinized with xylene, and rehydrated through an ethanol-water gradient. The Alexa-fluor 594 labelled probe for Gammaproteobacteria (GAM42a: 5′-GCC TTC CCA CAT CGT TT-3′) and the Alexa-fluor 488 labelled probe for Eubacteria (EUB338: 5′-GCT GCC TCC CGT AGG AGT-3′) were prepared at a concentration of 2.5 ng/μL in a hybridization solution (0.9 M NaCl, 0.1 M Trizma base, 0.1% SDS. and 30% formamide; pH 7.2). The sections were incubated with the probes at 37 °C for ≈18 hr in the dark. Slides were washed for 15 min in the hybridization solution without the probes, washed for an additional 15 min in a wash buffer (0.9 M NaCl and 0.1 M Tris base; pH 7.2), and were then placed in deionized water. Slides were mounted with fluoroshield with DAPI (Sigma, Markham, ON, Canada) and visualized and imaged using a T81X confocal microscope (Olympus, Richmond Hill, ON, Canada).

### Statistical analyses

All statistical analyses were performed using Statistical Analysis Software (SAS Institute Inc. Cary, NC). Continuous data was checked for normality and analyzed using the MIXED procedure of SAS. Where applicable (i.e. fecal *C. rodentium* densities, body weights, and feed intake), collection time was treated as a repeated measure; the appropriate covariance structure was utilized according to the lowest Akaike’s Information criterion. In the event of a main treatment event effect (P ≤ 0.050), the least squares means test was used to compare treatments within factors. Data is represented by mean ± standard error of then mean (SEM) and asterisks represent the following probability values: *P ≤ 0.050; **P ≤ 0.010; and ***P ≤ 0.001. Histopathologic measurement data were analysed using the Chi squared (NPAR1WAY) procedure of SAS.

## Additional Information

**How to cite this article**: Brown, K. *et al*. Antimicrobial growth promoters modulate host responses in mice with a defined intestinal microbiota. *Sci. Rep.* **6**, 38377; doi: 10.1038/srep38377 (2016).

**Publisher's note:** Springer Nature remains neutral with regard to jurisdictional claims in published maps and institutional affiliations.

## Figures and Tables

**Table 1:** qPCR primers used in this study.

Gene Target	Forward Primer (5′-3′)	Reverse Primer (5′-3′)	Annealing Temp (°C)	Amplicon Size	Source
*Ppia*	CTAAAGCATACAGGTCCTGGCA	ATGCCTTCTTTCACCTTCCCA	58	140	This study
*Hprt*	ACAGGCCAGACTTTGTTGGAT	ACTTGCGCTCATCTTAGGCT	58	150	This study
*Gusβ*	GCTCATCTGGAATTTCGCCG	CGGTTTCGTTGGCAATCCTC	58	149	This study
*Ifn-γ*	ACGGCACAGTCATTGAAAGC	TCTGGCTCTGCAGGATTTTCA	58	141	This study
*Il1β*	GTGTCTTTCCCGTGGACCTT	GGAGCCTGTAGTGCAGTTGT	58	151	This study
*Tnfα*	GATCGGTCCCCAAAGGGATG	GCTCCTCCACTTGGTGGTTT	58	98	This study
*ll6*	GACAAAGCCAGAGTCCTTCAGA	GTCTTGGTCCTTAGCCACTCC	58	158	This study
*Il17a*	GCAGCGATCATCCCTCAAAG	ACGTGGAACGGTTGAGGTAG	58	148	This study
*Il10*	ACAGCCGGGAAGACAATAAC	GGCAACCCAAGTAACCCTTA	58	174	This study
*Muc2*	AAAGACCACAACAGGGCCAA	GGTCCTGGTGGTCTCCAAAG	58	142	This study
*βd1*	GGCTGCCACCACTATGAAAACTC	GAGACAGAATCCTCCATGTTGAA	58	148	This study
*Saa1*	AAATCAGTGATGGAAGAGAGGC	CAGCACAACCTACTGAGCTA	58	178	This study
*Hp*	GCCATGGACTTTGAAGATGAC	CGAACCAAGTGCTCCACATA	58	74	This study
*Cp*	AACCCTGACACAAAAGGGAC	CCCTGCTTGGTGAGTAATCC	58	287	This study
*Hmgcl*	GTGAAGATGGCGTCAGTGAG	GGGGTGGGTACAATACTCTTT	58	167	This study
*Fabp1*	AAGTCAAGGCAGTCGTCAAG	TCAGTCACGGACTTTATGCC	58	76	This study
*Ctp1*	GGACTCCGCTCGCTCATT	TGCCATTCTTGAATCGGATGA	58	203	This study
*ApoA2*	TATAGCCCCTACCTCCAGTC	GCCTAGGAACAGTGCGATTC	58	73	This study
*Cramp*	TTCAACCAGCAGTCCCTAGA	TATCTGGATCCTCGTCCCCT	58	82	This study
*Il4*	CAGCAACGAAGAACACCACAG	GGCATCGAAAAGCCCGAAAG	58	153	This study
*Tgfβ*	GTCCAAACTAAGGCTCGCCA	CATAGTAGTCCGCTTCGGGC	58	152	This study
